# Lipopolysaccharide: Recent Advances in Its Biosynthesis and Controlling Cell Envelope Homeostasis

**DOI:** 10.3390/ijms26167705

**Published:** 2025-08-09

**Authors:** Satish Raina, Gracjana Klein

**Affiliations:** Laboratory of Bacterial Genetics, Gdansk University of Technology, Narutowicza 11/12, 80-233 Gdansk, Poland; satish.raina@pg.edu.pl

Typical Gram-negative bacteria, such as *Escherichia coli*, are diderms with two membrane bilayers separated by a periplasmic space containing a thin layer of peptidoglycan (PGN). Lipopolysaccharide (LPS) is the major component of the outer membrane (OM), whereas phospholipids (PL) are the main lipid component of the inner membrane (IM). The most defining feature of Gram-negative bacteria is the asymmetric nature of their OM, as they contain PL facing the inner leaflet and LPS located in the outer leaflet. LPS constitutes the major amphiphilic component of the OM. Bacteria, such as *E. coli* and *Salmonella*, contain approximately 2–3 × 10^6^ molecules of LPS that cover more than 75% of the OM [1]. The OM is essential for the viability of Gram-negative bacteria, with few exceptions. The maintenance of this asymmetry is crucial for bacterial cell envelope integrity, as it imparts a permeability barrier.

Structurally, LPS can be divided into three elements: the most conserved part is lipid A, to which a core polysaccharide is attached, which is further extended in smooth bacteria by the *O*-antigen. The biosynthesis of LPS begins with the acylation of UDP-Glc*N*Ac with *R*-3-hydroxymyristate derived from *R*-3-hydroxymyristoyl-ACP by LpxA and proceeds in a discontinuous manner. The second reaction of lipid A biosynthesis is catalyzed by LpxC [UDP-3-*O*-(*R*-3-hydroxymyristoyl)-*N*-acetylglucosamine deacetylase], constituting the first committed step in the LPS synthesis, as the equilibrium constant for the first reaction catalyzed by LpxA is unfavorable [2]. Following the deacetylation step, four additional enzymes, LpxD, LpxH, LpxB, and LpxK, act successively to generate the lipid IV_A_ intermediate [3]. In *E. coli*, all six enzymes are essential and, being unique to bacteria, are targets for new antimicrobials. Next, two Kdo residues are added to the lipid IV_A_ precursor, which enables the addition of secondary lauroyl and myristoyl chains, resulting in the synthesis of Kdo_2_-lipid A, which constitutes the hydrophobic anchor of LPS [4]. The hexaacylated lipid A part of LPS constitutes the endotoxin principal, as it is highly immunogenic and is recognized by the innate immune cell receptor TLR4/MD2-CD14 complex [5]. LPS can also be recognized in a TLR4/MD2-independent manner, involving recognition by caspases 4/5 in humans and 11 in mice [6].

The biosynthesis of the three main essential cell envelope components, PGN, LPS, and PL, is intricately linked. Thus, the enzymatic activities of glucosamine-6-phosphate synthase GlmS and deacetylase LpxC control the biosynthetic branch points using two key precursor molecules, UDP-*N*-acetyl-d-glucosamine (UDP-Glc*N*Ac) and *R*-3-hydroxymyristoyl acyl carrier protein (*R*-3-hydroxymyristoyl-ACP), respectively [4]. UDP-Glc*N*Ac is used as a substrate to initiate LPS synthesis via the LpxA/LpxC pathway and PGN biosynthesis by recruiting MurA/MurB enzymes. UDP-Glc*N*Ac is at the same time used as a key substrate for enterobacterial common antigen (ECA) biosynthesis. Bacteria synthesize fatty acids using the FASII system [7]. Fatty acid biosynthesis is highly regulated at this initial, rate-limiting step. The first committed step of fatty acid synthesis is the carboxylation of acetyl-CoA by the acetyl-CoA carboxylase enzyme complex (comprising AccA, B, C, and D components) to produce malonyl-CoA [8]. Next, malonyl-CoA is converted to malonyl-ACP by FabD. FabH initiates fatty acid synthesis by catalyzing the Claisen condensation of acetyl-CoA with malonyl-ACP to form acetoacetyl-ACP [9]. From here, the acyl-ACP is elongated by two carbons per cycle by elongation enzymes. Acyl-ACP of sufficient length is used by the acyltransferase system to produce phosphatidic acid from glycerol-3-phosphate. In *E. coli*, the acyltransferases PlsB and PlsC utilize long-chain acyl-ACPs to transfer acyl chains to the 1st and 2nd carbons of a glycerol phosphate moiety to generate phosphatidic acid [10]. The accumulation of acyl-ACPs feedback inhibits multiple enzymes involved in fatty acid biosynthesis, exerting another level of regulation [11,12]. Fatty acids comprise the acyl chains of glycerophospholipids, which are the major constituents of PL and the lipid A part of LPS. The first step in PL biosynthesis utilizes FabZ’s *R*-3-hydroxymyristoyl-ACP dehydratase activity. Thus, the fundamental common substrate of LpxC and FabZ, *R*-3-hydroxymyristoyl-ACP, constitutes an essential branch point in the biosynthesis of PL and the lipid A part of LPS. As acyl-ACPs are also used in the biosynthesis of PL and LPS, their availability could be a regulatory checkpoint to maintain a balance between PL and LPS levels. This ensures that the synthesis of LPS and PL is tightly co-regulated, as for the viability of bacteria, it has to be held at a nearly constant ratio of 0.15 to 1.0 [13].

Overall, LPS is highly heterogenous in chemical composition and can be present in different glycoforms within a bacterium, exhibiting further structural diversity across bacterial species [14]. Thus, variations exist in the lipid A part and the inner core due to changes in acyl chain length, changes in phosphorylation, and additional substitutions such as GlcN, rhamnose, GlcUA, and additional Kdo [15,16,17]. In *E. coli* and *Salmonella*, the most common non-stoichiometric substitutions are the incorporation of phosphoethanolamine (P-EtN) and 4-amino-4-deoxy-L-arabinose (Ara4N), which are critical for conferring resistance to cationic antimicrobial peptides (CAMPs). Variations in the Kdo group of Kdo_2_-lipid A have also been reported, including EptB-dependent modification of the second Kdo in *E. coli*, addition of galacturonic acid in *Rhizobium leguminosarum,* and, in some cases, hydrolysis of the outer Kdo [18,19,20]. Significantly, some lipid A modifications are important for survival under stress conditions and even for evading the TLR4-mediated immune response [21].

Recent discoveries: (i) Establishing cross-talk of cell envelope machineries: Underlining this intricate metabolic network of PGN, PL, and LPS is the generation of acetyl-ACP by malonyl-ACP decarboxylase MadA. MadA participates in the initial steps and allows FabH-independent fatty acid synthesis initiation, which defines the architecture of these elements [22]. Acetyl-ACP supports the initiation of fatty acid (FASII) and PL synthesis by entering FASII at the FabB/F-condensing enzyme step. Acetyl-ACP is also a substrate for GlmU, required for UDP-Glc*N*Ac synthesis, which acts as a metabolic precursor for PGN synthesis and for LpxA-mediated initiation of lipid A production.

(ii) Evidence of two major hubs that coordinate LPS and PL biosynthesis: Based on the toxic accumulation of LPS in the absence of LapB, it was shown that LapB participates in FtsH-mediated proteolysis of LpxC, and the lethal phenotype of either Δ*ftsH* or Δ*lapB* can be overcome in the presence of a gain-of-function mutation in *fabZ* [23]. Such a *fabZ* suppressor mutation acts by increasing the flux of acyl-ACPs towards PL, which are otherwise diverted to LPS by the stabilization of LpxC [24]. This interaction between LapB and FabZ was further reinforced by immunoprecipitations and, more importantly, the co-purification of LapB with LPS biosynthetic and transport proteins [23]. Thus, a broader function of LapB was proposed in acting as a scaffold for LPS assembly and regulating LpxC degradation. Such a scaffold-like function has been supported by additional observed interactions of LapB with LpxA and LpxD for lipid A biosynthesis and with FabZ in the fatty acid biosynthesis cycle [25]. Recently, it was shown that LapB interacts with LapD [26]. The analysis of the LapD interactome revealed that LapD-interacting partners involve fatty acid/PL biosynthetic enzymes such as AccD, MadA (FabY), and committed enzymes of FASII FabH, FabF, and FabB. Physical interactions have also been observed with PssA (phosphatidylethanolamine synthesis) and myristoyl transferase LpxM [26]. Suppressor analysis of Δ*lapD* revealed genetic interactions with *lpxM*, *clsA* (cardiolipin synthesis), and *acpP*. The interaction of LapD with the acetyl-CoA carboxylase enzyme complex and FASII enzymes and AcpP suggests that LapD could facilitate the coordinated regulation of fatty acid/PL and LPS and the existence of a broader hub. Some supporting evidence for the role of LapD in fatty acid regulation has recently been presented [27].

(iii) New structural insights into LPS assembly protein LapB and its function: As mentioned above, LapB is a multitasking protein that interacts with FtsH to mediate LpxC degradation. However, the mechanism by which LpxC is specifically recognized remains unclear. Recent studies have shown that LapB of *E. coli* uses its cytoplasmic TPR elements and C-terminal rubredoxin domain to interact with LpxC [28]. Another study in *Salmonella* showed that TPR2 of LapB facilitates LpxC binding and that the N-terminal transmembrane anchor, TPR1, TPRs 5–9, and the rubredoxin domain are not required for LapB-LpxC interaction under the experimental conditions used in these studies [29]. Thus, more studies are required to identify the specific amino acid residues in LapB that interact with FtsH and LpxC to regulate LPS levels. Interestingly, species-specific variations have also been reported for LapB. For example, in *Klebsiella pneumoniae*, the LapB structure reveals a distinct open-ring dimeric architecture, generated by TPR motif sliding, in contrast to the closed-ring dimer reported in the case of *E. coli* [30]. In *K. pneumoniae*, structural analysis of the dimeric form revealed interactions primarily via TPR5 of one monomer and TPR9 of the other [30].

(iv) New players in the regulation of LPS, regulatory control by PlsB, and requirement of alarmone ppGpp: Although most of the current studies on the regulation of LPS synthesis have centered around regulatory control via LpxC-FtsH/LapB and LapC, additional research shows that LpxA activity mediating the initial step in lipid A biosynthesis by itself might be one of the points of control. It has been shown that ppGpp inhibits LpxA activity [31]. Adding a new layer to the regulation of LPS, it has been shown that the essential GTPase ObgE interacts with LpxA. This was demonstrated based on the characterization of a dominant negative version of ObgE, which inhibits LpxA activity when bound to GTP [32]. Consistent with this regulation, suppressors that relieve the toxicity of ObgE were mapped to the *lpxA* gene [32]. A role for ppGpp in LPS biosynthesis was also previously shown in strains constitutively expressing the RpoE sigma factor, since Δ*rseA* ppGpp^0^ strains were unable to modify lipid A by Ara4N [19]. It has been known for a long time that the accumulation of ppGpp inhibits both de novo membrane PL biosynthesis and the incorporation of exogenous fatty acids into PL [33]. This inhibition was attributed to the accumulation of long-chain acyl-ACPs, which are substrates for the *plsB* gene product *sn*-glycerol-3-phosphate acyltransferase. In fatty acid/PL biosynthesis, key regulatory points are mediated by controlling the initiation rate-limiting step mediated by the carboxylation of acetyl-CoA by the ACC complex to produce malonyl-CoA, from which the malonyl group is transferred to *holo*-ACP by FabD to enter the elongation process. The second and probably more important is the regulation of the first step in PL synthesis, catalyzed by the PlsB enzyme, which synthesizes lysophosphatidic acid from long-chain ACP and *sn*-glycerol-3 phosphate. Based on experimental and modeling studies, it has been shown that PL flux is regulated primarily via post-translational control of PlsB enzyme activity [34]. It has been shown that PlsB activity controls the concentration of the LPS precursor C14:0-OH-ACP, linking fluxes into the PL and LPS synthesis pathways, as altering PL synthesis by adjusting either PlsB or ACC activity would also vary LPS synthesis [34].

Although the main regulation of LpxC occurs post-transcriptionally up to a 20-fold level [35], at the transcriptional level, SoxS induction can increase *lpxC* gene expression, which causes cell lysis [36]. Consistent with these findings, the accumulation of SulA in the absence of Lon protease in nitrofurantoin-treated bacteria increased LPS synthesis and transporter-related gene expression, resulting in increased LPS production [37]. Another post-transcriptional regulator that requires further study is the GcvB sRNA-mediated control of LpxC. Overexpression of GcvB sRNA represses the accumulation of LpxC and suppresses the lethality of LapAB absence [38]. Interestingly, LpxC can also be subjected to proteolysis independent of FtsH-LapB by the HslVU protease in *E. coli* [39]. HslVU-mediated proteolysis of LpxC may be particularly important for regulating LPS synthesis at high temperatures, as the activity of this protease is enhanced under these growth conditions.

(v) New insights in LPS transport machinery: It is well established that the essential inner membrane protein MsbA plays a pivotal role in flipping the LPS precursor from the cytoplasmic to the periplasmic leaflet of the IM. LPS flipping involves the binding of LPS to the interior binding site and a conformational change from an inward- to an outward-facing conformation [40,41]. These studies further revealed that MsbA has two LPS-binding sites: one located in the central cavity and the other at a membrane-facing exterior site [40,41]. More recent studies have further probed these MsbA-LPS interactions using native mass spectrometry with Kdo_2_-LA precursor as a substrate [42]. Kdo_2_-lipid A can tune the selectivity of MsbA for ATP over ADP [42]. Another interesting recent study addressed the interplay between TMDs and NBDs of MsbA by examining the involvement of two intracellular loops, coupling helix 1 and 2 (CH1, CH2) [43]. The authors observed significant chemical shift changes within both CH1 and CH2 upon substrate binding, in the ATP hydrolysis transition state, and upon MsbA inhibitor binding [43].

Once LPS is flipped to the periplasmic leaflet of the IM, it is further extracted by the Lpt protein machinery, which comprises seven essential Lpt proteins (LptB_2_FGCADE) that function together as a transenvelope bridge. Four of the proteins, LptB_2_FGC, form an ABC transporter that extracts LPS from the IM [44]. After extraction from the IM, LPS is transferred from the periplasmic domain of the IM-anchored LptC to LptA in the periplasm and subsequently delivered to the OM protein complex LptDE [45]. In the last decade, several studies have shown that the proper functioning of the Lpt bridge is the assembly and maturation of LptD, which requires disulfide bond-forming proteins (Dsbs) [46]. Recently, another lipoprotein, LptM, was shown to facilitate the rearrangement of disulfide bonds in LptD, adding a new layer of regulation [47]. Latest structural studies of LptDE-LptM suggest that LptM translocates to the OM via LptDE, in a manner similar to LPS transport [48].

(vi) Targeting LPS biosynthesis and transport for antibiotic discovery: The emergence of multidrug resistance in ESKAPE pathogens that include Gram-negative bacteria, such as *Acinetobacter* and *Pseudomonas*, poses a new challenge for human health. As LPS is essential in the majority of Gram-negative bacteria and several proteins involved in LPS biogenesis, assembly, and transport are unique and required for bacterial growth, they have become important targets for the discovery of new antibiotics. Thus, the discovery of inhibitors such as CHIR090, which targets LpxC, mediating the first committed step in LPS biosynthesis, proved vital in this direction. However, many LpxC inhibitors exhibit unexpected cardiovascular toxicity, hampering their use. Recently, a slow, tight-binding LpxC inhibitor, LPC-233, with low picomolar affinity displaying good activity against a wide range of Gram-negative clinical isolates in vitro has been described, which holds significant promise for clinical use [49]. Research has also been directed toward finding inhibitors of other enzymes involved in LPS biosynthesis and transport, including LpxA, LpxD, LpxH, MsbA, LapC, and proteins of the Lpt complex [50,51,52,53]. To this end, inhibitors of MsbA structure-based drug development have been analyzed. One series of MsbA inhibitors are quinoline-based molecules that prevent proper NBD closure to suppress ATP hydrolysis and substrate transport [54,55]. Another series of MsbA inhibitors are tetrahydrobenzothiophene-based molecules that force a collapsed inward-facing state, thereby disrupting NBD-TMD communication and yielding higher basal ATPase activities [56,57]. Another approach is to target Lpt proteins to prevent LPS transport to the OM. This is best illustrated by the characterization of the natural product thanatin and the synthetic peptide murepavadin, which have been shown to be effective against *E. coli* and *Pseudomonas aeruginosa,* respectively. Thanatin inhibition of LPS transport has been proposed because of its binding to the N-terminal domain of LptA, which normally interacts with LptC [58]. Murepavadin is a peptidomimetic antibiotic that blocks the function of *P. aeruginosa* lipopolysaccharide transporter LptD [59]. However, the identification of mutations in *lpxL* and *pmrB* genes that confer resistance to murepavadin and colistin suggests a potentially additional mechanism of its target in *P. aeruginosa* [60]. As interest in the identification of new targets continues to grow, a new class of macrocyclic peptides targeting the Lpt system of *Acinetobacter baumannii* has led to the identification of zosurabalpin, which has shown promise in clinical trials and has been found to inhibit the release of LPS from the LptFG cavity [61,62].

(vii) Cellular response to LPS defects and structural alterations: Recent studies have shown that any gross structural alterations, inhibition of LPS synthesis or assembly or transport, or changes in PL levels trigger cellular responses similar to other envelope stress conditions [4]. *E. coli* cell envelope stress response was initially studied as a response to defects in periplasmic protein folding or imbalance in OMP composition, which is regulated by the extracytoplasmic function sigma factor RpoE and the two-component system CpxA/R [63,64]. As OMP maturation defects also depend on LPS composition, LPS defects are also sensed by the RpoE and CpxA/R systems [65]. RpoE is an alternative sigma factor that is highly induced in the absence of LapB or when LapC is dysfunctional due to an imbalance in LPS vs. PL amounts [23,39]. The RpoE counterpart (AlgU) in *P. aeruginosa* was recently found to be induced upon treatment with murepavadin, indicating a role of the envelope stress response in bacterial tolerance to this antibiotic [66]. In *Salmonella enterica* serovar Typhimurium, specific LPS defects (absence of LapB) or truncation of LapC have been shown to activate the Rcs two-component system [29].

In continuation of the above-mentioned discoveries in this Special Issue, six research articles were published that cover the structural diversity in LPS composition, techniques for LPS quantification in terms of biological activity, regulation of LPS by LapC and identification of new components that regulate LpxC, targeting OM asymmetry by antimicrobial peptides affecting PL migration, and structural alterations in the LPS core that impact the immune response in pathogenic bacteria.

In the manuscript by Dardelle et al., the authors first described the analysis of the complexity and diversity of LPS structures in selected well-studied bacterial species [67]. The suitability of various techniques, including visualization of differences in various chemotypes by SDS-PAGE analysis, structural analysis by MALDI-MS, and chemical methods for quantification, was evaluated. The authors describe in detail the structural variability of LPS with examples of two *Yersinia* strains grown at different temperatures and LPSs from other genera, particularly variations in the acylation of the lipid A part of LPS. Furthermore, the impact of different LPS structures obtained under different bacterial growth conditions on selective LPS detection methods, such as Limulus Amoebocyte Lysate (LAL), HEK-blue TLR-4, LC-MS^2^, and MALDI-MS, is described. As expected, LPSs responded differently in these assays. This was attributed to the properties of lipid A structures, including the length and number of acyl chains, the presence of phosphate groups, and their possible substitutions. The impact of structural differences on the toxicity of LPS was shown by cytokine induction and MAT or HEK-Blue hTLR4 signalization. However, the LAL assay did not respond to LPS modifications and did not differentiate between toxic and non-toxic LPS forms, although it exhibited the highest sensitivity. Additionally, HEK-Blue hTLR4 was shown to be more specific for measuring potential LPS toxicity, and its use along with the LAL assay is suggested.

In continuation of the theme of diversity of LPS structure, another manuscript describes the structural properties of the LPS from the rhizosphere bacterium *Ochrobactrum quorumnocens* T1Kr02 [68]. Analysis of the fatty acid composition of LPS revealed the presence of 3-hydroxytetradecanoic, hexadecenoic, octadecenoic, and 27-hydroxyoctacosanoic acids. The authors analyzed the structure of its *O*-polysaccharide (PS-1). The remarkable feature of this polysaccharide is that it contains d-Fuc*p* and d-Fuc*f* residues, with 50% *O*-acetylation of d-Fuc. These findings are significant because d-Fuc monosaccharides are relatively rare components of bacterial polysaccharides. This study also examined the physicochemical properties of its LPS in aqueous solution using the dynamic light-scattering method. The results showed that LPS particles had a diameter of 72.2 ± 3.6 nm and a zeta-potential of −21.5 ± 0.7 mV, which can be a useful marker for future comparison of strains from related bacteria. Regarding biological activity, it was shown that LPS from *O. quorumnocens* strain T1Kr02 improves the development of microplants in vitro and hence could be used to increase the efficiency of microclonal propagation of potato.

In the manuscript by Li et al., the authors evaluated the impact of variants of antimicrobial peptides cathelicidins (As-CATHs) obtained from *Alligator sinensis* on the membranes of Gram-negative bacteria [69]. The authors used As-CATH4, AS4-1, AS4-5, and AS4-9 peptides with decreasing charges but increasing hydrophobicity and evaluated them by performing molecular dynamics simulations. All four variant peptides disrupted the structures of the IM of bacteria, with AS4-9 exhibiting the highest antibacterial activity. As the AS4-9 peptide has moderate charges and hydrophobicity, it significantly perturbs the membrane structure by squeezing the lipids in the proximal leaflet and can even extract them from the membrane at sufficiently high peptide concentrations. The authors postulate that the binding of AS4-9 promotes PL flipping from the inner leaflet to the outer leaflet, changing OM asymmetry. In this manner, the AS4-9 peptide could bind to PL and extract it from the membrane. This mode of action could promote their antibacterial activity and allow the future construction of novel peptide-based antibiotics.

In an extension of previous work on LapC- and LapD-mediated regulation of LpxC, Maniyeri et al. used new strategies to identify additional players that could mediate LpxC regulation [70]. In this study, the authors exploited the temperature sensitivity (Ts) and membrane permeability defects of *lapC* mutant bacteria expressing only the N-terminal domain of LapC. Isolation and characterization of multicopy suppressors that corrected the Ts phenotype identified specific factors predicted to be involved in maintaining homeostasis between LPS and PL biosynthesis. Thus, one group comprises genes encoding LPS assembly factor (LapD), acyl-carrier protein AcpP, known to shuttle fatty acids for lipid A and fatty acid biosynthesis, subunit of acetyl-CoA carboxylase enzyme AccB, and outer membrane phospholipase PldA, which removes mislocalized PL from the outer leaflet of the OM. Another set of genes encoded either transcription factors (*dksA*, *marA*, and *srrA*) or the *gnsA* gene, whose product is predicted to regulate the amounts of saturated versus unsaturated fatty acids, or could act indirectly (*yfgM*, *yceJ*). It was further shown that overexpression of *srrA* and *marA* genes fully restored LpxC levels, and DksA-mediated suppression was only to a milder extent. However, interestingly, overexpression of *acpP* or *accB* did not restore either LpxC or LPS levels in *lapC* mutant bacteria and was proposed to act by balancing PL and LPS amounts. Overexpression of *acpP* could inhibit PL synthesis at the PlsB level, and *accB* could disrupt the assembly of the ACC complex, thereby reducing fatty acid synthesis and helping in balancing PL amounts to reduced amounts of LPS in *lapC* mutant bacteria. It was further shown that MarA suppression requires the functionality of the Mla system involved in the maintenance of lipid asymmetry, providing a rationale for the requirement of MarA in the regulation of LPS. A similar analysis revealed that SrrA-mediated suppression was contingent on the presence of cardiolipin synthase A encoded by the *clsA* gene. Confirming the important roles of these players, this study also showed that *srrA*, *marA*, and *pldA* became essential when LapC was dysfunctional. As overexpression of the *lapD* gene can overcome the Ts phenotype of *lapC* mutant bacteria in an LpxC-independent manner, it was shown that both the N-terminal IM anchor and the last 32 C-terminal residues are critical for LapD function. Concerning DksA, which is a well-characterized transcriptional factor (TF) [71] that exhibits peptidyl *cis*/*trans* isomerase activity (PPIase) [72], it was shown that both TF and PPIase activities of DksA are required for its role in regulating LpxC levels. Thus, in this study new players in LpxC regulation, such as MarA, SrrA, and GnsA, were identified, and the roles of the acyl-carrier protein AcpP and acetyl-CoA carboxylase complex in maintaining a balance between LPS and PL were proposed.

The manuscript by Pérez-Ortega et al. examined the effect of mutations that alter LPS structure on the activation of Toll-like receptor (TLR4) in the pathogenic bacteria *Bordetella pertussis* [73]. The authors showed that dephosphorylation of lipid A in *B. pertussis* through the heterologous production of the phosphatase LpxE from *Francisella novicida* does not affect TLR4-stimulating activity. The authors rationalized these results due to the presence of phosphorylated Kdo in the inner core of *B. pertussis* LPS, which could mediate the interaction with the TLR4–MD2 receptor complex. Thus, the Kdo region in the inner core was modified either mutationally or by the heterologous expression of bifunctional *E. coli* Kdo transferase, as KdtA in *B. pertussis* is essential. Interestingly, the presence of KdoII in *B. pertussis* LPS impaired the substitution of the outer core with the terminal trisaccharide with a variable loss of the secondary C_14_ chain at the 2′ position of lipid A. The authors next identified the *eptB* gene, whose product mediates the non-stoichiometric substitution of Kdo-bound phosphate with P-EtN, and furthermore inactivated this gene on the chromosome. Based on these genetic modifications, it was shown that the expression of *kdtA*_Ec_ increased the TLR4-activating capacity of *B. pertussis* cells. In contrast, *eptB* inactivation reduced TLR4 activation. Moreover, inactivation of *eptB* in the LpxE_Fn_-producing strain further reduced its TLR4-stimulating capacity. Hence, the authors concluded that *eptB* inactivation, in combination with LpxE_Fn_ production, could be a promising approach for developing whole-cell vaccines against pertussis with reduced endotoxicity.

Another manuscript addressed whether radio-detoxified LPS in the form of an environmental aerosol spray can be used as a prophylactic agent in pollen-induced allergic reactions [74]. The authors applied LPS in an aerosol form, using conditions that mimic conditions on farms, where LPS levels are higher than those in inner-city homes prior to the allergen challenge. A preventative effect of pre-exposure to the radiation-detoxified form of LPS (RD-LPS) on pollen-induced allergy in six-week-old mice was observed. Next, the effects of aerosolized native LPS (N-LPS) and RD-LPS were compared with those on human monocyte-derived dendritic cells (moDCs). Results from such experiments revealed that RD-LPS-exposed moDCs had a higher Th_1_-polarizing capacity than moDCs exposed to N-LPS. Examination of cytokine levels showed that exposure to RD-LPS resulted in significantly lower levels in the case of the pro-inflammatory cytokine IL-1β and the anti-inflammatory cytokine IL-10 compared to N-LPS. As IL-10 is known as a global suppressor of immune responses, the authors argue that IL-10 production of RD-LPS-exposed moDCs might be due to a greater increase in β-hydroxymyristic acid in the RD-LPS preparations than in N-LPS by free fatty acids. These results are in agreement with the notion that free fatty acids have immunomodulatory properties and can influence the outcomes of immune responses in the body. These observations led the authors to suggest that aerosolized, non-toxic LPS applied in early life for the renaturation of urban indoor environments could be a strategy to prevent Th_2_-mediated allergies in childhood.

As is evident from the review of recent advances presented here, the research in LPS continues to provide major new insights in terms of its regulation, structural diversity, transport, and assembly and is of high importance for the development of new antimicrobial compounds, given its essentiality in Gram-negative bacteria.
